# Convergent validity and inter-rater reliability of a lower-limb multimodal physical function assessment in community-dwelling older adults

**DOI:** 10.3389/fragi.2023.1196389

**Published:** 2023-06-20

**Authors:** Myles W. O’Brien, Heather F. Neyedli, Laurent Bosquet, Brianna Leadbetter, Alex Smith, Francois Gallant, Pamela Tanguay, Mathieu Bélanger, Said Mekari

**Affiliations:** ^1^ School of Physiotherapy (Faculty of Health), Department of Medicine (Faculty of Medicine), Dalhousie University, Halifax, NS, Canada; ^2^ Geriatric Medicine Research, Dalhousie University & Nova Scotia Health, Halifax, NS, Canada; ^3^ Division of Kinesiology, School of Health and Human Performance, Faculty of Health, Dalhousie University, Halifax, NS, Canada; ^4^ Laboratoire MOVE (UR20296), Université de Poitiers, Faculté des Sciences Du Sport, Poitiers, France; ^5^ School of Kinesiology, Faculty of Professional Studies, Acadia University, Wolfville, NS, Canada; ^6^ Department of Family Medicine, Université de Sherbrooke, Sherbrooke, QC, Canada; ^7^ Centre de Formation Médicale Du Nouveau-Brunswick, Université de Sherbrooke, Moncton, NB, Canada

**Keywords:** timed up-and-go, lower limb, convergent validity, physical function, healthy aging

## Abstract

**Introduction:** Lower-limb physical function declines with age and contributes to a greater difficulty in performing activities of daily living. Existing assessments of lower-limb function assess one dimension of movement in isolation or are not time-efficient, which discourages their use in community and clinical settings. We aimed to address these limitations by assessing the inter-rater reliability and convergent validity of a new multimodal functional lower-limb assessment (FLA).

**Methods:** FLA consists of five major functional movement tasks (rising from a chair, walking gait, stair ascending/descending, obstacle avoidance, and descending to a chair) performed consecutively. A total of 48 community-dwelling older adults (32 female participants; age: 71 ± 6 years) completed the FLA as well as timed up-and-go, 30-s sit-to-stand, and 6-min walk tests.

**Results:** Slower FLA time was correlated with a slower timed up-and-go test (*ρ* = 0.70), less sit-to-stand repetitions (*ρ* = −0.65), and a shorter distance in the 6-min walk test (*ρ* = −0.69; all, *p* < 0.001). Assessments by two raters were not different (12.28 ± 3.86 s versus 12.29 ± 3.83 s, *p* = 0.98; inter-rater reliability *ρ* = 0.993, *p* < 0.001) and were statistically equivalent (via equivalence testing). Multiple regression and relative weights analyses demonstrated that FLA times were most predicted by the timed up-and-go performance [adjusted *R* ^*2*^ = 0.75; *p* < 0.001; raw weight 0.42 (95% CI: 0.27, 0.53)].

**Discussion:** Our findings document the high inter-rater reliability and moderate-strong convergent validity of the FLA. These findings warrant further investigation into the predictive validity of the FLA for its use as an assessment of lower-limb physical function among community-dwelling older adults.

## 1 Introduction

Physical function declines with age, predisposing older adults to adverse health effects (Metti et al, 2018) and may result in an inability to carry out instrumental and basic activities of daily living (Mayhew et al, 2020). Specifically, tasks such as rising from a chair, climbing the stairs, and avoiding obstacles are more difficult with age. A quantification of patients’ physical function is necessary to determine level of independence and burden of health conditions and to develop individualized exercise plans. While individual assessments of physical functions exist (Hetherington-Rauth et al, 2022), they are typically administered in controlled environments that do not reflect real-life movements. Valid functional assessments that are time-effective may prove useful for clinicians and exercise professionals.

Optimal mobility is largely determined by an effective coordination of the neural, muscular, and cardiovascular systems. Established assessments of lower-limb function in older adults include the timed up-and-go (TUG) (Rose et al, 2002), 30-s sit-to-stand (STS) (Yee et al, 2021), and 6-min walk test (6MWT) (Rikli and Jones, 1998; Hamilton and Haennel, 2000). The objective of these physical function tests are to assess isolated movement components, with different aspects across the TUG (velocity, agility, and dynamic balance), STS (lower-limb physical function and muscular strength), and 6MWT (gait speed and cardiorespiratory fitness). Free-living activities are composed of multiple compound movements. Time-efficient assessments that consider the holistic nature of physical function may be a superior model for quantifying older adults’ lower-limb physical function in contrast to isolated assessments. This idea has been adopted in alternative tests, such as the Dynamic Gait Index (Herman et al, 2009), that uses one overall test broken into eight facets of walking gait. However, this assessment takes ∼15 min and requires relatively extensive instructions for patients, reducing its utility in clinical or community settings. Alternative assessments that are quick and easy for practitioners to implement, while also being reflective of real-world movements for older adults, are needed. Accordingly, combining the aspects of gait and other functional tasks, such as transitioning from sitting to standing within a single, short test, may be useful for clinicians and researchers to assess older adult patients’ physical function. In addition to putting forth alternative protocols to expand providers’ “toolbox” of physical assessments, it is imperative that such tests produce consistent results among assessors.

The purpose of our study was to assess the inter-rater reliability and convergent validity of a new multimodal functional lower-limb assessment (FLA) in community-dwelling older adults. The test consists of five major functional movements: rising from a chair, walking gait, stair ascending/descending, obstacle avoidance, and descending to a chair. Each of the five movements is performed consecutively, and the overall task is timed. We explored whether the FLA performance concurred with the results from other individual assessments of physical function (convergent validity) and established the FLA inter-rater reliability.

## 2 Materials and methods

### 2.1 Participants

A convenient sample of 49 community-dwelling older adults (17 male and 32 female participants) was recruited to participate in the study from the Acadia Active Aging and Acadia Active for Life program at Acadia University. All participants were at least 60 years of age. All participants did not require ambulatory devices (e.g., walkers) and reported no major musculoskeletal injuries. The Get Active Questionnaire was used to screen participants prior to the study. It is meant to clear the generally healthy population to physical activity and identify any chronic disease or diagnostics that might hinder participation in physical activity (Petrella et al, 2018).

Participants were informed of the methods and study procedures verbally and in writing before providing written informed consent. All protocols and procedures conformed to the Declaration of Helsinki and were approved by the Acadia University Research Ethics Board.

### 2.2 Experimental design

Prior to testing, heart rate (via radial pulse), blood pressure (via sphygmomanometer and brachial cuff), and anthropometrics (i.e., height and weight via a stadiometer) were determined. The participants attended a single session when each of the FLA, TUG, STS, and 6MWT was administered. One male participant did not complete the STS test. Sufficient rest (i.e., 5–10 min) was allotted between each test to minimize fatigue. The order of the functional assessments was randomized.

### 2.3 Multimodal functional lower-limb assessment

**FIGURE 1:**
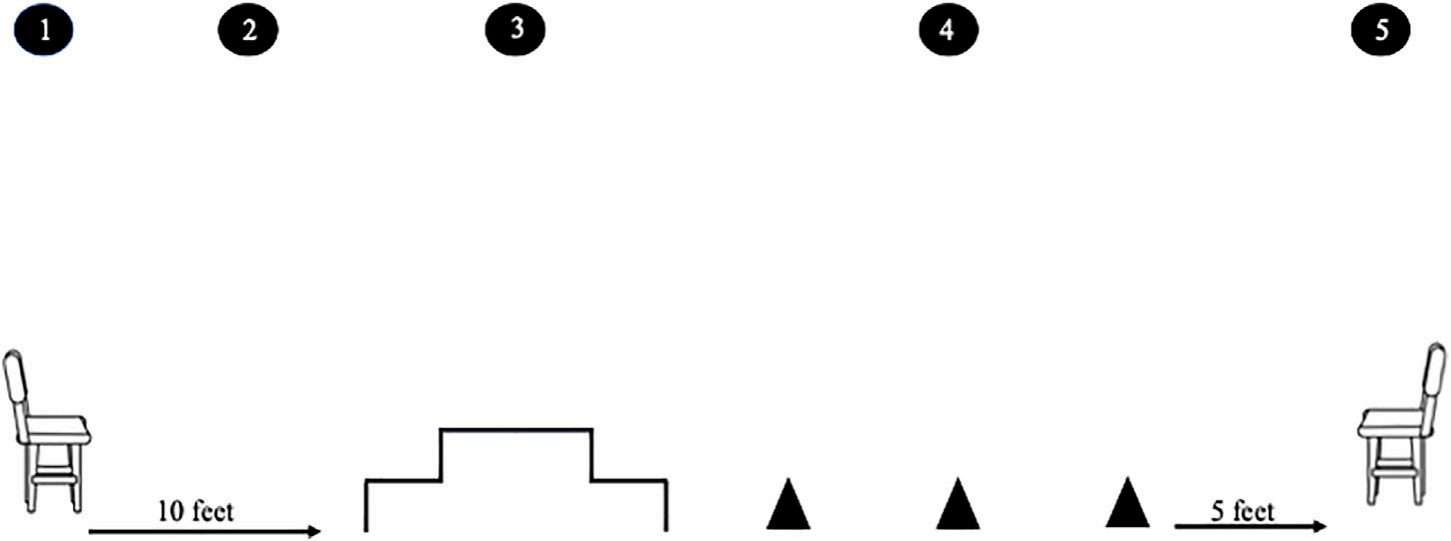
FLA consisted of five sequential functional movements: 1) standing up from a chair, 2) walking 10 feet in a straight line at a normal pace to a set of two standard modified Canadian Aerobic Fitness Test steps positioned back-to-back, 3) walking up the two steps on one side and down the two steps on the other side, encouraging the participants to carry this out at a pace that they would normally navigate the stairs in their day-to-day life, 4) weaving in and around a set of three cones placed 5 feet apart, and 5) walking 5 feet, turning around, and sitting down on a chair.

The FLA consisted of five sequential functional movements: 1) rising from a chair, 2) walking gait, 3) stair ascending/descending, 4) obstacle avoidance, and 5) descending to a chair. The participants started in a seated position in a chair without arm rests. They stood up (#1) and walked 10 feet in a straight line at their normal pace (#2) to a set of two standard modified Canadian Aerobic Fitness Test (Weller et al, 1995) steps positioned back-to-back. The participants walked up the two steps on one side and down the two steps on the other side (#3) and were encouraged to carry out this at a pace they would normally navigate stairs in their day-to-day life. Then, they weaved in and around a set of three cones placed 5 feet apart (#4). Lastly, they walked 5 feet, turned around, and sat down on a chair without arm rests at the other end of the course (#5) (see Figure 1).

The timer was started on the first forward movement of the participant’s back and was stopped when the participant was fully seated on the chair at the end. The goal was to perform the test as comfortably as possible and to perform each task as safely as possible at their self-determined normal movement speed; this was explained to the participants prior to the task. A back-to-back two-step staircase over a three-step staircase was selected because the standard modified Canadian Aerobic Fitness Test steps may be more readily available to researchers or exercise professionals. These staircases do not have a handrail. The participants were also provided with an explanation and a demonstration of the test and were invited to ask any questions (Table 1). A familiarization trial was not provided, and only one attempt was permitted. The test was timed by two assessors independently during the same participant’s attempt of the FLA. The exact same instructions were provided to each participant. Individual assessor scores were compared for inter-rater reliability (see Section 2.5). Each functional movement was scored on a three-point scale including the scores of 0/2 (severe impairment), 1/2 (moderate impairment), or 2/2 (normal) (see Supplemental File S1). An overall score out of 10 was calculated (FLA score). Raters’ times were averaged for regression analyses.

**TABLE 1:** Verbal instructions to be given during the demonstration to participants.

Administer location	Verbal instruction
Seated in the start chair	“Stand up and walk to the stairs.”
At the base of the stairs	“Walk up the two stairs and then down the two stairs.”
After descending the stairs	“Weave through the cones.”
After weaving through the cones	“Turn around and sit down safely on the chair.”

### 2.4 Other functional assessments

We chose to compare our proposed FLA with commonly implemented physical assessments, including the TUG, STS, and 6MWT, which were conducted using standardized procedures (Rikli and Jones, 1998; Jones et al, 1999; Rose et al, 2002). For each participant, instructions and a brief demonstration was provided. For the TUG test, each participant stood up from a chair, walked 8 feet around a cone, and descended back to the chair (Rose et al, 2002). The recorded time between raters was averaged for the TUG test. For the STS test, the participants were instructed to cross their arms over their chest and stand up, straightening their legs fully, and then to sit back down far enough to touch their buttocks to the chair as many times as they could safely within a 30-s time frame (Jones et al, 1999). Only full repetitions from sitting to standing were counted. Any repetitions that the participants did not touch the chair on the downward phase or did not straighten their legs on the upward phase were not counted toward their score. The 6MWT was completed according to standardized procedures provided by the American Thoracic Society (ATS Statement: guidelines for the six-minute walk test, 2002). It was conducted in an open gym, and the course was marked by red cones placed 30 m apart. The cumulative distance covered over the 6 min was recorded to the nearest centimeter (Rikli and Jones, 1998).

### 2.5 Determining FLA inter-rater reliability

Statistical analyses for reliability were conducted using SPSS (version 28.0, IBM Corp., Armonk, NY, United States). Statistical significance was set at p <0.05. All data are presented as means ± SD.

Statistical guidelines for conducting validity and reliability analyses for activity-monitor studies were adopted for our analytical strategy (Welk et al, 2019). The rater’s times were determined to be non-parametric via the Shapiro–Wilk test (both, *p* < 0.001). As such, a Wilcoxon signed-rank test, Spearman’s rank correlation, and mean absolute error test (Rater 1–Rater 2) were conducted for the FLA time between raters. FLA scores (out of 10) were compared via a weighted kappa.

We also conducted equivalence testing to determine whether the two raters provided statistically equivalent measures (O’Brien, 2021). For this, the confidence interval (CI) method was used, where 90% CIs of the differences in means (i.e., rater 1–rater 2) are tested to be within a specified equivalence zone. We calculated the minimum equivalence zone threshold as a proportion of SD (Lakens, 2017), with thresholds of low (1 SD), medium (0.5 SD), high (0.25 SD), and very high equivalence (0.15 SD) (O’Brien, 2021). Furthermore, the Bland–Altman method of differences was used to visually compare FLA times between raters (Ludbrook, 2010). This allowed to assess the presence of a fixed bias (i.e., mean difference not equal to “0”) with a one-sample *t*-test and proportional bias (i.e., the amount of difference is dependent on the magnitude of the average values) via a linear regression between the difference and mean of the rater’s time.

### 2.6 Relationship between functional assessments

Regression analyses were conducted using R.3.3.1. Spearman’s rank correlations were conducted to explore the relationships between FLA, TUG, STS, and 6MWT performances. Multiple regression analysis was conducted to understand the relationships between the FLA and other established measurements of the lower-limb physical function simultaneously. The FLA time was modeled as a continuous outcome, and the TUG, STS, and 6MWT were inserted as independent variables in a single step. The score (out of 10) was not used given the few individuals who scored <9 out of 10 on the assessment, as indicated in the Results section.

To examine which of the TUG, STS, or 6MWT test was most strongly related to FLA, we conducted relative importance analysis in conjunction with regression analysis (Johnson and LeBreton, 2004). This allowed the estimation of the raw weight that each variable contributes to the overall model. The statistical significance of the weights was determined via 10,000 replication bootstrapping, with statistical significance denoted by 95% confidence intervals not encompassing zero.

## 3 Results

One male participant was removed across all analyses because he was an influential point (Cook’s score: 6.7) along the predictors (TUG: 17.5 s—4.8 SD away from the group mean; STS: 3 reps—2.2 SD away from the group mean; 6MWT: 122.0 m—3.6 SD away from the group mean) and was an outlier in the outcome variable (FLA time: 55 s—5.8 SD away from the group mean) performing worse in all tests compared to the rest of the participants in the sample. The remaining participants’ characteristics and outcomes of each functional test are presented in Table 2. For the FLA, most participants scored 10/10 (31/48, 65%), with some scoring 9 (12/48, 25%) and few scoring <9 (5/48, 10%).

**TABLE 2:** Participant characteristics and physical function outcomes.

Variable	Participant (*n* = 48)
Participant characteristics	
Age (years)	71 ± 6 (60–89)
Sex (male and female)	16 and 32
Height (cm)	168 ± 8 (149–185)
Weight (kilograms)	75.1 ± 14.1 (46.8–108.5)
Body mass index (kg/m^2^)	26.6 ± 4.1 (17.0–39.4)
Resting heart rate (beats/min)	73 ± 11 (52–96)
Systolic blood pressure (mmHg)	125 ± 11 (108–152)
Diastolic blood pressure (mmHg)	77 ± 7 (64–96)
Physical function outcomes	
Timed up-and-go (seconds)	6.6 ± 1.6 (4.0–12.1)
30-s sit-to-stand (repetitions)	18.9 ± 6.8 (8–35)
6-min walk test (distance in meters)	550 ± 102 (220–730)
Multimodal functional assessment score (out of 10)	9.6 ± 0.8 (6.5–10.0)
Multimodal functional assessment time (seconds)	12.3 ± 3.8 (7.8–27.0)

Data are presented as proportions or means ± SD (range).

### 3.1 Inter-rater reliability

Spearman’s rank-order correlations demonstrated a strong relationship between times of rater 1 and 2 (*ρ* = 0.993; *p* < 0.001), and a Wilcoxon signed-rank test determined that the times between the raters were not different (12.28 ± 3.86 s versus 12.29 ± 3.83 s, *p* = 0.98). The mean absolute difference was 0.16 ± 0.15 s between raters. Consistent with this, the minimum equivalence zone required for the raters to be statistically equivalent was 0.01 SD, indicating a very high statistical equivalence. There were few differences in raters’ scores (39/49 were the same and 10/49 differed by 1 out of 10), with a weighted kappa of 0.72 (95% CI: 0.55–0.89, *p* < 0.001).

**FIGURE 2:**
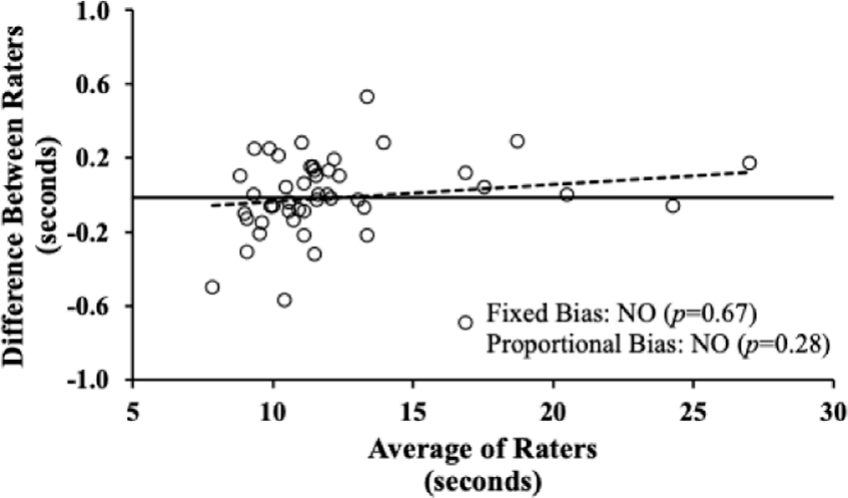
Bland–Altman plot comparing the differences in the functional locomotion assessment overall time between rater 1 and rater 2 (y-axis) versus the mean of the two raters (x-axis). Circles represent 49 individual data points; the black solid line signifies the mean difference; the broken black line depicts the slope (β-coefficient) of the relationship. Neither a fixed nor a proportional bias was observed.

Bland–Altman analyses (Figure 2) demonstrated that neither a fixed bias (mean difference: −0.01, 95% CI = −0.08–0.05 s, *p* = 0.67) nor a proportional bias was present (slope: 0.009, 95% CI = −0.008–0.026, *p* = 0.28).

### 3.2 Relationships between assessments

**FIGURE 3:**
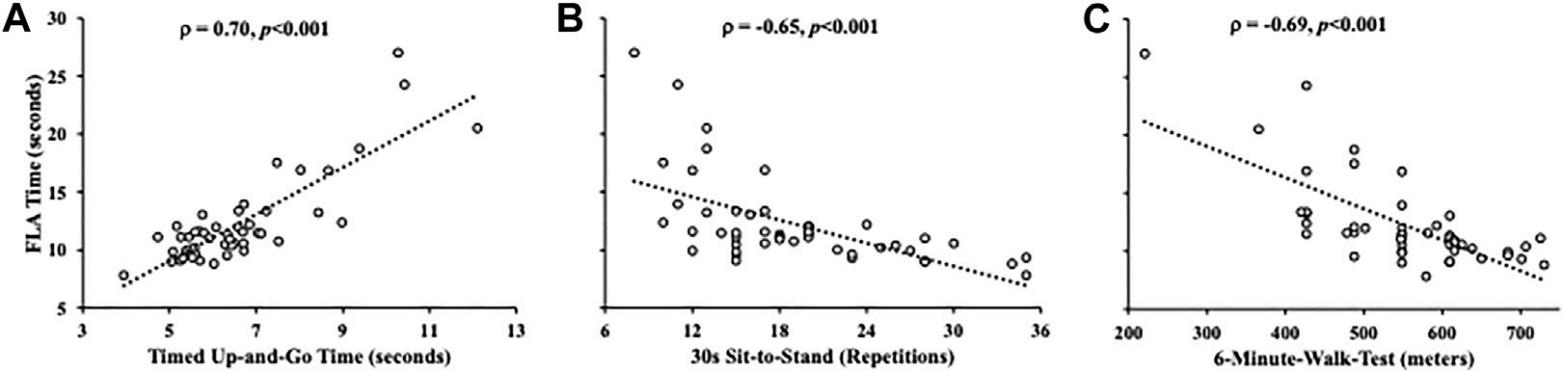
Scatterplots depicting the relationship between the time on the functional locomotion assessment with the timed up-and-go **(A)**, 30-s sit-to-stand **(B)**, and 6-minute walk test **(C)**. The relationships were determined via Spearman’s rank correlations (ρ).

Time on the FLA was positively associated with TUG time (*ρ* = 0.70, *p* < 0.001) (Figure 3A) but was negatively associated with STS repetitions (*ρ* = −0.64, *p* < 0.001) (Figure 3B) and 6MWT distance (*ρ* = −0.69, *p* < 0.001) (Figure 3C) via Spearman’s correlations.

The 75% of the variance in FLA time could be explained by the combination of the three other functional assessment tests, F (3, 44) = 47.1, *p* < 0.001, *R* ^*2*^ = 0.76, and adjusted *R* ^*2*^ = 0.75. Given that the TUG, 6MWT, and STS were all correlated, a relative importance analysis was used to assess the contribution each of these variables make to predict FLA time while accounting for collinearity [24]. As presented in Table 3, all three predictors had statistically significant relative weights linking them with FLA time, as indicated by 95% bootstrapped confidence intervals. TUG had the largest relative weight (0.42), followed by the 6MWT (0.21) and the STS performance (0.13).

**TABLE 3:** Multiple regression model predicting time on the multimodal functional assessments from the timed up-and-go, 30-s sit-to-stand, and 6-min walk test.

Predictor	Unstandardized β (95% CI)	Standard error	Standardized β	*p*-value	Relative raw weight (95% CI)
TUG (time)	1.50 [1.03, 1.98]	0.234	0.62	<0.001	0.42 [0.27, 0.53]
STS (repetitions)	−0.07 [−0.18, 0.03]	0.054	−0.13	0.19	0.13 [0.07, 0.18]
6MWT (distance)	−0.008 [−0.017, −0.001]	0.004	−0.23	0.04	0.21 [0.11, 0.34]
Intercept	8.57 [2.12, 15.0]	3.203	—	0.01	—

Data are presented as unstandardized β [95% confidence intervals]. The strength of the model is presented. *If the relative weights of 95% confidence intervals did not encompass zero, then they are statistically significant. TUG, timed up-and-go; STS, sit-to-stand; 6MWT, 6-minute walk test.

## 4 Discussion

We present a multimodal FLA in a sample of community-dwelling older adults. Our initial observations provide encouraging findings in support of the relatively short FLA as a single test of lower-limb physical function among older adults. Specifically, FLA performance was similar to the performance of common assessments of lower-limb physical function, most particularly TUG time and 6MWT. Furthermore, we observed a very high inter-rater reliability with statistically equivalent FLA times and scores between raters.

Locomotion is among the most fundamental aspects of health (Brown et al, 2014; Paluch et al, 2022). While the included functional assessments (TUG, STS, and 6MWT) are staple assessments in determining lower-limb physical function with well-established clinical validity (Rikli and Jones, 1998; Hamilton and Haennel, 2000; Rose et al, 2002; Yee et al, 2021), the isolated nature of these tests limits their representation of daily life. The moderate-strong relationships between scores on these tests and the FLA performance indicated a reasonable level of convergent validity. While we did not establish the clinical utility of the FLA directly, this indirect assessment of validity is encouraging and warrants research assessing the clinical relevance and predictive capabilities of the FLA to differentiate the disease status and/or mobility. The FLA performance was the most predicted by the TUG time. TUG is a marker of gait performance and can discriminate older adults who have experienced a fall versus those who have not (Rose et al, 2002). Additionally, the TUG test is sensitive to change following nutritional supplement (Bischoff et al, 2003) and physical activity (Kerse et al, 2008) interventions among older adults. Given the relationship between the FLA and TUG, this may indirectly indicate that the FLA may also provide clinically relevant information regarding fall risks, but future works are needed to determine the utility of the FLA and whether the performance of the FLA is sensitive to interventions or change in mobility among older adults. Given that the TUG performance was the primary weight of the FLA time, a faster FLA time may most likely be dictated by a better dynamic balance and mobility and, albeit to a lesser extent, by muscular strength (STS performance) and muscular endurance (6MWT performance). Given the requirements of the FLA that includes climbing stairs and navigating through stationary cones, it is unsurprising that the attributes required for the TUG test are consistent between these assessments. It is plausible that the dynamic FLA may be a more thorough assessment of the lower-limb function given the variety of tasks, which are relatively more challenging than a simple TUG test. However, this remains to be thoroughly determined, and an evaluation of whether the additional administrative requirements of the FLA (e.g., using cones and stairs) provide more clinically relevant results than those of the TUG test alone is needed. Furthermore, the battery of TUG, STS, and 6MWT requires ∼7 min of movement plus explanations, set-up, and rest time between tests. The FLA only requires ∼0.5 min in a single test, negating the need for rest between tests, which is one of the strengths of this study. Altogether, we introduce a novel multimodal FLA assessment, and our moderate-strong convergent validity encourages further research on its ability to predict diseases or mobility concerns.

Introducing the FLA by examining the inter-rater reliability and its correlation with existing assessments in a sample of older adults is reflective of the initial presentation of the TUG test in the literature (Mathias et al, 1986). Of note, the sample size of the present study (*n* = 48) was similar to the initial presentation of the TUG test (*n* = 40) (Mathias et al, 1986), but nevertheless, the objective of this study was to test this assessment and determine whether two scores provide consistent results. Additionally, the inter-rater reliability for our FLA was very high, which is similar to the inter-rater reliability for the TUG test (correlation coefficients: >0.93) in community-dwelling and hospitalized older adults (Kristensen et al, 2019). Using a battery of statistical tests (Welk et al, 2019), we demonstrated that two observers scoring the FLA produce statistically equivalent times. Ensuring that the two raters produce consistent outcomes is an important consideration in proposing a new physical function assessment. Such convergent validity and high inter-rater reliability are encouraging given the increased complexity of the FLA (i.e., comprised of five movements) in comparison to the existing lower-limb assessments. The FLA may be a suitable alternative to existing lower-limb functional assessments, with the present study laying a foundation for further studies of this assessment tool.

Our test is specific to the sample of interest and may not be feasible for less healthy adults with mobility limitations that prevent stair climbing, for example. While this single test may not address all of the limitations of the other tests, it could provide an additional option for clinicians and researchers to use, as it fits with the objective of their care and research objectives. For example, the Short Physical Performance Battery is a well-established series of lower-limb assessments designed for older adults is predictive of disability and mortality (Guralnik et al, 1994; Pavasini et al, 2016). The FLA provides more options for physical assessments directed toward an older population that combines multiple aspects of lower-limb function and may even more so reflect the complexities of everyday movements compared to a single isolated test. Therefore, the addition of the FLA alongside the Short Physical Performance Battery may provide unique but complementary information regarding lower-limb physical function. The findings of this initial study prompt further research on clinical populations with and without walking aids. Certainly, the introduced FLA is specific to physical function of the lower limb and does not reflect upper-limb physical function. However, it has been opined that lower-limb function is more likely to decline to a greater extent with age (Ditroilo et al, 2010), as it predicts mobility (Vasunilashorn et al, 2009) and the primary contributor to functional independence (Batista et al, 2014). It is appreciated that more space and tools (i.e., a set of stairs and cones) may be needed to conduct the FLA in comparison to TUG and STS tests. Two-step stairs may already be available in laboratories and clinics that have conducted modified Canadian Aerobic Fitness Tests, which are designed to be relatively mobile and can be set up easily. Furthermore, the setup of equipment and distance between equipment requires ∼40 feet (or ∼12 m) of a straight hallway or gym clearance space, which is less than the space required to conduct a 6MWT. Studying modified versions of the FLA that use more/less stepping distance or alternative staircases may be of interest for researchers/clinics interested in fitting this assessment into their space requirements. While inter-rater reliability was observed to be high, it is unclear how much visit-to-visit (or day-to-day) variability is exhibited by the FLA. Future studies into the longer-term reliability and predictive capabilities of the FLA performance on markers of health (e.g., presence of disease) or mobility are warranted. It is unclear whether the FLA time or score is a superior outcome of the test, but the narrow range of scores (90% scored 9 or 10) substantiates the need for further studies on populations facing challenges performing lower-limb function tests. Alternatively, a scoring system that grades each task by incorporating more options that reward the ease of the movement (e.g., very easy, easy, neutral, difficult, very difficult, and cannot complete) may be a better assessment.

In conclusion, we introduced a new, brief assessment of lower-limb physical functions that is associated with more traditional clinical assessments but reflects more ecologically valid day-to-day physical behaviors. We established the FLA moderate-strong convergent validity and a high inter-rater reliability, but further research studies on the utility of this assessment within clinical, research, and/or community settings are warranted.

## Data Availability

The original contributions presented in the study are included in the article/Supplemental Materials; further inquiries can be directed to the corresponding author.

## References

[B1] Ats Statement (2002). Guidelines for the six-minute walk test. Am. J. Respir. Crit. Care Med. 166, 111–117. 10.1164/ajrccm.166.1.at1102 12091180

[B2] Batista F. S. Gomes G. A. de O. D’Elboux M. J. Cintra F. A. Neri A. L. Guariento M. E. (2014). Relationship between lower-limb muscle strength and functional independence among elderly people according to frailty criteria: A cross-sectional study. Sao Paulo Med. J. 132, 282–289. 10.1590/1516-3180.2014.1325669 25054965 PMC10496748

[B3] Bischoff H. A. Stähelin H. B. Dick W. Akos R. Knecht M. Salis C. (2003). Effects of vitamin D and calcium supplementation on falls: A randomized controlled trial. J. Bone Min. Res. 18, 343–351. 10.1359/jbmr.2003.18.2.343 12568412

[B4] Brown J. C. Harhay M. O. Harhay M. N. (2014). Walking cadence and mortality among community-dwelling older adults. J. Gen. Intern. Med. 29, 1263–1269. 10.1007/s11606-014-2926-6 24934147 PMC4139514

[B5] Ditroilo M. Forte R. Benelli P. Gambarara D. De vito G. (2010). Effects of age and limb dominance on upper and lower limb muscle function in healthy males and females aged 40–80 years. J. Sports Sci. 28, 667–677. 10.1080/02640411003642098 20397097

[B6] Guralnik J. M. Simonsick E. M. Ferrucci L. Glynn R. J. Berkman L. F. Blazer D. G. (1994). A short physical performance battery assessing lower extremity function: Association with self-reported disability and prediction of mortality and nursing home admission. J. Gerontol. 49, M85–M94. 10.1093/geronj/49.2.M85 8126356

[B7] Hamilton D. M. Haennel R. G. (2000). Validity and reliability of the 6-minute walk test in a cardiac rehabilitation population. J. Cardiopulm. Rehabil. 20, 156–164. 10.1097/00008483-200005000-00003 10860197

[B8] Herman T. Inbar-Borovsky N. Brozgol M. Giladi N. Hausdorff J. M. (2009). The Dynamic Gait Index in healthy older adults: The role of stair climbing, fear of falling and gender. Gait Posture 29, 237–241. 10.1016/j.gaitpost.2008.08.013 18845439 PMC2709498

[B9] Hetherington-Rauth M. Magalhães J. P. Alcazar J. Rosa G. B. Correia I. R. Ara I. (2022). Relative sit-to-stand muscle power predicts an older adult’s physical independence at age 90 beyond that of relative handgrip strength, physical activity and sedentary time. *Am. J. Phys. Med. Rehabil.* Publ. Ah 101, 995–1000. 10.1097/PHM.0000000000001945 35034060

[B10] Johnson J. W. LeBreton J. M. (2004). History and use of relative importance indices in organizational research. Organ. Res. Methods 7, 238–257. 10.1177/1094428104266510

[B11] Jones C. J. Rikli R. E. Beam W. C. (1999). A 30-s chair-stand test as a measure of lower body strength in community-residing older adults. Res. Q. Exerc. Sport 70, 113–119. 10.1080/02701367.1999.10608028 10380242

[B12] Kerse N. Peri K. Robinson E. Wilkinson T. Randow M. V. Kiata L. (2008). Does a functional activity programme improve function, quality of life, and falls for residents in long term care? Cluster randomised controlled trial. BMJ 337, a1445. 10.1136/bmj.a1445 18845605 PMC2565754

[B13] Kristensen M. T. Bloch M. L. Jønsson L. R. Jakobsen T. L. (2019). Interrater reliability of the standardized Timed up and Go Test when used in hospitalized and community‐dwelling older individuals. Physiother. Res. Int. 24, e1769. 10.1002/pri.1769 30657232

[B14] Lakens D. (2017). Equivalence tests: A practical primer for t tests, correlations, and meta-analyses. Soc. Psychol. Personal. Sci. 8, 355–362. 10.1177/1948550617697177 28736600 PMC5502906

[B15] Ludbrook J. (2010). Confidence in altman-bland plots: A critical review of the method of differences. Clin. Exp. Pharmacol. Physiol. 37, 143–149. 10.1111/j.1440-1681.2009.05288.x 19719745

[B16] Mathias S. Nayak U. S. Isaacs B. (1986). Balance in elderly patients: The “get-up and go” test. Arch. Phys. Med. Rehabil. 67, 387–389. 3487300

[B17] Mayhew A. J. Griffith L. E. Gilsing A. Beauchamp M. K. Kuspinar A. Raina P. (2020). The association between self-reported and performance-based physical function with activities of daily living disability in the Canadian longitudinal study on aging. Journals Gerontol. Ser. A 75, 147–154. 10.1093/gerona/glz122 PMC6909898 31081885

[B18] Metti A. L. Best J. R. Shaaban C. E. Ganguli M. Rosano C. (2018). Longitudinal changes in physical function and physical activity in older adults. Age Ageing 47, 558–564. 10.1093/ageing/afy025 29546417 PMC6693378

[B19] O’Brien M. W. (2021). Implications and recommendations for equivalence testing in measures of movement behaviors: A scoping review. J. Meas. Phys. Behav. 4, 1–10. 10.1123/jmpb.2021-0021

[B20] Paluch A. E. Bajpai S. Bassett D. R. Carnethon M. R. Ekelund U. Evenson K. R. (2022). Daily steps and all-cause mortality: A meta-analysis of 15 international cohorts. Lancet Public Heal 7, e219–e228. 10.1016/S2468-2667(21)00302-9 PMC9289978 35247352

[B21] Pavasini R. Guralnik J. Brown J. C. di Bari M. Cesari M. Landi F. (2016). Short physical performance battery and all-cause mortality: Systematic review and meta-analysis. BMC Med. 14, 215. 10.1186/s12916-016-0763-7 28003033 PMC5178082

[B22] Petrella A. F. M. Gill D. P. Petrella R. J. (2018). Evaluation of the get active questionnaire in community-dwelling older adults. Appl. Physiol. Nutr. Metab. 43, 587–594. 10.1139/apnm-2017-0489 29342366

[B23] Rikli R. E. Jones C. J. (1998). The reliability and validity of a 6-minute walk test as a measure of physical endurance in older adults. J. Aging Phys. Act. 6, 363–375. 10.1123/japa.6.4.363

[B24] Rose D. J. Jones C. J. Lucchese N. (2002). Predicting the probability of falls in community-residing older adults using the 8-foot up-and-go: A new measure of functional mobility. J. Aging Phys. Act. 10, 466–475. 10.1123/japa.10.4.466

[B25] Vasunilashorn S. Coppin A. K. Patel K. V. Lauretani F. Ferrucci L. Bandinelli S. (2009). Use of the short physical performance battery score to predict loss of ability to walk 400 meters: Analysis from the InCHIANTI study. Journals Gerontol. Ser. A Biol. Sci. Med. Sci. 64A, 223–229. 10.1093/gerona/gln022 PMC2655026 19182232

[B26] Welk G. Bai Y. Lee J. M. Godino J. O. B. Saint-Maurice P. F. Carr L. (2019). Standardizing analytic methods and reporting in activity monitor validation studies. Med. Sci. Sports Exerc. 51, 1767–1780. 10.1249/MSS.0000000000001966 30913159 PMC6693923

[B27] Weller I. M. R. Thomas S. G. Gledhill N. Paterson D. Quinney A. (1995). A study to validate the modified Canadian aerobic fitness test. Can. J. Appl. Physiol. 20, 211–221. 10.1139/h95-015 7640647

[B28] Yee X. S. Ng Y. S. Allen J. C. Latib A. Tay E. L. Abu Bakar H. M. (2021). Performance on sit-to-stand tests in relation to measures of functional fitness and sarcopenia diagnosis in community-dwelling older adults. Eur. Rev. Aging Phys. Act. 18, 1. 10.1186/s11556-020-00255-5 33419399 PMC7791746

