# Supplementary material for: Convergent validity and inter-rater reliability of a lower-limb multimodal physical function assessment in community-dwelling older adults
Source: Front Aging. 2023 Jun 20;4:1196389. doi: 10.3389/fragi.2023.1196389 (PMC10318151; doi:10.3389/fragi.2023.1196389)
Supplement: Supplementary file 1 [file DataSheet1.docx]

**SUPPLEMENTAL FILE 1**

**Multimodal Functional Lower-Limb Assessment**

**Rise from Chair**

(2) Normal: Rises from chair with no obvious imbalance;

(1) Moderate Impairment: Rises from chair with obvious imbalance OR uses ambulatory device;

(0) Severe Impairment: Must be assisted from chair.

Notes:________________________________________________________________________

______________________________________________________________________________

**Gait**

(2) Normal: Normal gait pattern;

(1) Moderate Impairment: Abnormal gait pattern OR uses ambulatory device;

(0) Severe Impairment: Severe gait deviations or obvious imbalance.

Notes:________________________________________________________________________

______________________________________________________________________________

**Stair Climbing**

(2) Normal: Alternating feet, no loss of balance;

(1) Moderate Impairment: Obvious loss of balance OR two feet to a stair;

(0) Severe Impairment: Needs assistance OR Unable to climb.

Notes:________________________________________________________________________

______________________________________________________________________________

**Obstacle Avoidance**

(2) Normal: Able to walk around cones safely without changing gait speed, no obvious imbalance.

(1) Moderate Impairment: Able to avoid cones, but must slow down and adjust steps to clear cones OR uses ambulatory device.

(0) Severe Impairment: Unable to clear cones OR requires assistance.

Notes:________________________________________________________________________

**Lowering to Chair**

(2) Normal: Lowers to chair safely with no obvious imbalance;

(1) Moderate Impairment: Lowers to chair with obvious imbalance OR uses ambulatory device;

(0) Severe Impairment: Must be assisted to chair.

Notes: ________________________________________________________________________

Task Total:_______________ Time:_______________
